# Equal palliative care for patients with COPD? A nationwide register study

**DOI:** 10.1080/03009734.2019.1586803

**Published:** 2019-04-23

**Authors:** Ingela Henoch, Peter Strang, Claes-Göran Löfdahl, Ann Ekberg-Jansson

**Affiliations:** a Department of Research and Development, Angered Local Hospital, Gothenburg, Sweden;; b The Sahlgrenska Academy, Institute of Health and Care Sciences, University of Gothenburg, Sweden;; c Department of Oncology-Pathology, Karolinska Institutet, Stockholm, Sweden;; d Stockholms Sjukhem Foundation´s Research and development unit, Stockholm, Sweden;; e University of Lund, Sweden;; f COPD Center, Institute of Medicine, Sahlgrenska University Hospital, University of Gothenburg, Sweden;; g Department of Research and Development, Region Halland, Sweden;; h The Sahlgrenska Academy, Institute of Medicine, University of Gothenburg, Sweden

**Keywords:** Palliative care, chronic obstructive pulmonary disease, symptoms, advance care planning, register study

## Abstract

**Background:** Although chronic obstructive pulmonary disease (COPD) is a life-limiting disease with a significant symptom burden, the patients are more often referred to nursing homes (NH), than to specialist palliative care (SPC) at the end of life (EOL). This study aimed to compare patients with COPD in SPC with those in NH and to compare the care provided.

**Methods:** A national register study was carried out where the Swedish National Airway Register and the Swedish Register of Palliative Care were merged. COPD patients who died in NHs or short-term facilities were included in the NH group (*n* = 415) and those who died in SPC were included in the SPC group (*n* = 355). Demographic and clinical variables were included from the Swedish National Airway Register and variables concerning EOL care from the Swedish Register of Palliative Care.

**Results:** Symptom prevalence was similar in NHs and SPC, but symptom assessment (32% vs 20%), symptom relief medication (93-98% in SPC vs 74-90% in NH), EOL discussions (88% vs 66%), and bereavement support (94% vs 67%) were more likely in SPC (in all comparisons *p* < 0.001). Younger age and co-habiting increased the probability of dying in SPC (*p* < 0.001).

**Conclusion:** Despite similar symptom prevalence, older persons are more likely to be referred to NHs. If applying a palliative care philosophy in NHs, routine symptom assessment and prescription of rescue medication for frequent symptoms, would be more likely. Promoting advance care planning and EOL discussions at an earlier stage would result in more prepared patients and families.

## Introduction

Chronic Obstructive Pulmonary Disease (COPD), a progressive and life-limiting disease, is predicted to be the third-leading cause of death by the year of 2020, globally (1). Despite troublesome symptoms in advanced stages, COPD still receives considerably less attention in specialized palliative care (SPC) services, than does cancer (2,3).

Prominent symptoms in COPD include an increasing number of exacerbations with pneumonia (4), anxiety (5), and depression (4,6), which is associated with impaired prognosis (7). The patients also exhibit increasing dyspnea, partly due to respiratory failure (4,8–10), decreased exercise capacity (4), low BMI (8), dry mouth (10), cough (10), sleep problems (10), and pain (10). Moreover, COPD patients are often affected by comorbidities (11), such as heart failure, thrombo-embolic episodes, osteoporosis and renal failure.

In COPD, physical symptoms and psychological wellbeing interact, as breathlessness and anxiety often appear in a cycle; breathlessness triggers anxiety, and anxiety triggers breathlessness (12). Pharmacologic respiratory treatment of the disease can positively influence severe dyspnea, and, in the most severe cases, when combined with opioids, can decrease breathlessness (13,14). Severe COPD also has psychological, social and existential consequences, such as social isolation, loss of hope, and a struggle to maintain meaning in life (12).

In a review, HRQoL in palliative care patients was related to symptom burden and health care issues, but also to cognitive, emotional, social, and spiritual aspects, and to issues about personal autonomy, and preparedness (15). Most of these aspects are highly relevant to patients with COPD and their families. The latter aspects, i.e., awareness and preparedness, are central to palliative and end of life (EOL) care. However, there are several barriers to promoting these. In a survey of respiratory physicians, only one-third regularly discussed palliative care issues with their patients (16). Given the gradual progression and the prognostic uncertainty of these individuals (17), health care professionals might be unaware of the patient with COPD being in the palliative phase, which may result in limited planning and provision of palliative care (18). The consequences of this non-awareness and organizational barriers, may result in a reluctance to initiate EOL discussions or advance care planning (18).

As COPD is a life-limiting disease, associated with a significant symptom burden and comorbidities, these patients should be considered as candidates for specialist palliative care (SPC) at the EOL, as SPC home care and in-patient care units have special attention on symptom control, as well as on psychosocial and existential support to patients and their families. In Sweden, SPC is provided by palliative home care teams or by palliative in-patient care units. Palliative home care teams often co-operate with hospitals which provide disease-modifying treatments whereas the home care team is responsible for symptom control, support, management of nutrition, follow-up visits and so on. Typically, the length of care is for some months. The palliative in-patient care facilities are mainly used in the dying phase for the last 2–3 weeks of life. Obviously, SPC units are experts on symptom control and support. Still, we know that a considerable number of COPD patients are referred to nursing homes. For the above reasons, the aim of this research was to compare patients diagnosed with COPD treated in specialized palliative care with those in nursing homes, regarding the following research questions:

- What are the characteristics of patients diagnosed with COPD who received specialized palliative care compared to patients cared for in nursing homes?
- What are the characteristics of the care provided to patients diagnosed with COPD in specialized palliative care and in nursing homes?
- Is the provided care equal in quality and equity - regardless of place of care?
- What predicts health care admission to specialized palliative care or nursing home care in the EOL care of patients with COPD?

## Methods

This is a register study including two national quality registers, the Swedish National Airway Register and Swedish Register of Palliative Care (SRPC). The Swedish National Airway Register is a register of patients diagnosed with either COPD or asthma, where registrations of each patient visit were made by healthcare professionals in out-patient units, mostly primary healthcare and only a minority, 14%, in specialized pulmonary clinics. Registrations concerned demographic, clinical, and patient-reported variables. In the present study, the last registrations were identified. In the SRPC, healthcare professionals at the unit where the patients had died made registrations of demographic and clinical characteristics of the patients, place of death, and some characteristics of the EOL care, and symptoms in the last week of life.

### Sample

An extract from the Swedish National Airway Register was obtained in 2016 and a total of 20 668 patients diagnosed with COPD were identified, with each attending at least one visit. Of these, deceased patients were identified through the Swedish Tax Agency, and we found 3113 patients who had died. The identities of the deceased patients were cross-referenced with the identities of patients in the SRPC and 1842 of the deceased patients were also found there. The two databases were then merged. The patients whose place of death was a nursing home (*n* = 279) or short-term service for older people (*n* = 136) were identified and merged to one group called the “nursing home group” (*n* = 415). The patients who died in specialized in-patient palliative care (*n* = 252) or in their homes with support from specialized palliative home health care (*n* = 103) were merged to one group called the “specialized palliative care group” (*n* = 355).

### Variables

The variables from the Swedish National Airway Register related to demographic characteristics, i.e., age, gender, and social situation. Clinical characteristics related to stage of COPD according to the GOLD stages (1), number of exacerbations the last 12 months, number of hospitalizations due to COPD in the last 12 months, FEV_1_% of predicted value, comorbidities, and exercise capacity, measured by the number of days per week that the patient had been physically active. Patient-reported variables comprised smoking habits, divided into non-smokers, ex-smokers, and still smokers. Dyspnea was measured by the Modified Medical Research Council (mMRC) dyspnea scale (19), which is a patient-rated, single-item scale where severity of the dyspnea experience is reported, ranging from 0, corresponding to “Not troubled by breathlessness except on strenuous exercise”, to 4, corresponding to “breathless when dressing or undressing”. HRQoL was measured by the Clinical COPD Questionnaire (CCQ) (20), a patient-rated questionnaire with ten items, measuring dyspnea at rest, dyspnea during physical activities, cough, phlegm, how concerned the patient is about the dyspnea; and how the dyspnea had limited the patient’s activities, i.e., strenuous physical activities, moderate physical activities, daily activities, and social activities. All items are scored by the patient from 0, “never”, to 6, which corresponds with “almost all the time”. A mean score of 4.0–6.0 is interpreted as a large or very large impact on HRQOL; 2.0–3.9 corresponds with moderate impact on HRQOL, and 0.0–1.9, no or small impact on HRQOL. Minimal clinical important difference in the CCQ was found to be 0.4 (21). In later registrations, health status was measured by the COPD Assessment Test (CAT) (22). The CAT consists of eight items relating to cough, phlegm, pressure on chest, dyspnea on exertion, limitations in performing activities, risks due to lung condition, sleep quality, and level of energy. All items are scored by the patients from 0, “without problems”, to 5, which corresponds with “severe problems”. All scores of the items are added together to obtain a single score ranging from 0 to 40.

From the SRPC, the following variables were retrieved: whether death was expected; whether the patient was able to express his/her wishes the last part of life; whether the place of death was preferred by the patient; whether anyone was present at death; and whether there had been any EoL discussion about the impending death with either the patient or the family; whether the family was invited to participate in the after-death dialogue; and whether members of other professions were consulted. Data about symptoms during the latter part of life were also retrieved, such as presence of pressure ulcers, symptom assessments, symptoms prevalence during the last week of life, assessments made, prescribed medications, and data on whether the symptom was alleviated. The following symptoms: pain, rattle, nausea, anxiety, dyspnea, and delirium, were registered.

### Data analysis

Descriptive statistics were used for describing the sample and their registered treatments, with mean values and standard deviations (SD) calculated for continuous variables and numbers and percentages of the total sample for categorical variables. *T*-test was used to make comparisons between continuous variables. Relationships between dichotomous categorical experience and treatment variables and age, gender, and social situation were analyzed with the Mantel-Haenszel Chi-square test.

In order to explore predictors of place of death, we performed bivariate logistic regression analyses with a dependent variable with nursing homes as place of death scored as 0 and specialized palliative care as place of death scored as 1. Independent variables were: age; gender; social situation; FEV1% of predicted value; number of exacerbations in the last 12 months; number of hospital admissions due to COPD in the last 12 months; BMI; exercise capacity; smoking; HRQoL measured by CCQ; dyspnea measured by mMRC; and comorbidities. Secondly, independent variables that significantly predicted the dependent variable with *p* < 0.20 in the bivariate analyses were entered in the multivariate stepwise logistic regression analyses with the same dependent variable.

## Results

In total, 355 and 415 patients admitted to specialized palliative care (SPC) and nursing homes (NH), respectively, were included in the study.

### Differences between patient groups

Patients receiving SPC were significantly younger (mean 73.9 vs 79.3 years, respectively) and more often co-habiting than NH patients (Table 1). Patients in specialized palliative care were also to a larger extent still smokers (30.4% vs 23.0%, *p* < 0.001). NH patients had significantly more comorbidities than patients in SPC, i.e., a significantly higher proportion were affected by heart failure, ischemic heart disease, hypertension, diabetes, osteoporosis, and depression (*p* = 0.036 – 0.001, see Table 1).

**Table 1.** Differences in patients who died in either specialized palliative care or in nursing homes (*n* = 770).

	Patients receiving specialized palliative care, in-patient or in their own home (*n* = 355)	Nursing home and short-term facilities (*n* = 415)	*p*-value, diff between sp pall care and nursing home
	Mean (SD)	Mean (SD)	
Time between last visit and death, days	662.6 (518.5)	618.9 (521.4)	0.24
Demographic variables			
Age	73.9 (8.0)	79.3 (7.7)	<0.001
	*n* (%)	*n* (%)	
Gender			
Men	168 (47.3%)	204 (49.2%)	0.61
Women	187 (52.7%)	211 (50.8%)	
Social situation:			
Living alone	48 (32.9%)	68 (56.7%)	<0.001
Co-habiting	98 (67.1%)	52 (43.3)	
Clinical variables	*n* (%)	*n* (%)	
Stage of COPD			0.28
I: (80-100% of predicted FEV1)	11 (4.3%)	6 (2.7%)	
II: (50-79% of predicted FEV1)	86 (33.6%)	73 (32.7%)	
III: (30-49% of predicted FEV1)	103 (40.2%)	98 (43.9%)	
IV: (0-29% of predicted FEV1)	56 (21.9%)	43 (19.3%)	
	Mean (SD)	Mean (SD)	
Number of exacerbations in the last 12 months	1.37 (2.06)	1.24 (2.04)	0.48
Number of hospitalizations in the last 12 months	0.62 (1.30)	0.76 (1.82)	0.32
FEV% of predicted value	43.50 (18.42)	43.52 (16.65)	0.99
Exercise capacity	2.42 (2.79)	1.79 (2.58)	0.012
Patient -reported variables	*n* (%)	*n* (%)	
Smoking:			
Non-smoker	15 (4.7%)	40 (13.5%)	<0.001
Have quit smoking	205 (64.9%)	188 (63.5%)	
Still smoking	96 (30.4%)	68 (23%)	
	Mean (SD)	Mean (SD)	
Dyspnea	2.61 (1.30)	2.80 (1.23)	0.113
HRQoL (CCQ)	2.29 (1.34) (*n* = 150)	2.41 (1.22) (*n* = 124)	0.42
HRQoL (CAT)	17.95 (7.61) (*n* = 59)	17.78 (6.90) (*n* = 45)	0.91
Comorbidity	*n* (%)	*n* (%)	
Heart failure	45 (18.4%)	92 (36.7%)	<0.001
Ischemic heart disease	60 (24.4%)	102 (39.8%)	<0.001
Stroke	18 (8.9%)	21 (11.5%)	0.39
Hypertension	118 (45.0%)	173 (60.3%)	<0.001
Diabetes	35 (13.3%)	62 (24.7%)	0.001
Osteoporosis	39 (17.9%)	57 (26.9%)	0.025
Depression/anxiety	65 (25.0%)	88 (33.3%)	0.036
Lung cancer	19 (9.5%)	17 (8.9%)	0.84
Alpha-1-antitrypsin deficiency	3 (1.7%)	0	0.094

*T*-test was used for comparisons between continuous variables, Chi 2 for comparisons between categorical, variables.

### Characteristics and content of care: symptom management

There were no significant differences in symptom prevalence between SPC and NHs for any of the symptoms of pain, death rattle, nausea, anxiety, dyspnea, or delirium (Table 2). Neither was there a difference as regards the prevalence of pressure ulcers at admission or at death.

**Table 2.** Characteristics of symptom experience, assessment, and management registered in specialized palliative care and in nursing homes.

Palliative care characteristics	Patients receiving specialized palliative care (*n* = 355)	Nursing home and short-term facilities (*n* = 415)	*p*-value, diff between sp pall care and nursing home
Pressure ulcer on admission			0.53
No pressure ulcer	285 (85.3%)	292 (85.1%)	
Grade 1	21 (6.3%)	19 (5.5%)	
Grade 2	17 (5.1%)	14 (4.1%)	
Grade 3	7 (2.1%)	11 (3.2%)	
Grade 4	4 (1.2%)	7 (2.0%)	
Pressure ulcer at death			0.88
No pressure ulcer	242 (71.6%)	255 (72.9%)	
Grade 1	43 (12.7%)	41 (1.7%)	
Grade 2	37 (10.9%)	25 (7.1%)	
Grade 3	11 (3.3%)	17 (4.9%)	
Grade 4	5 (1.5%)	12 (3.4%)	
Symptoms or assessments during the last week of life			
Mouth health assessment	253 (78.6%)	193 (62.9%)	<0.001
Symptom assessment other than pain	101 (32.0%)	67 (20.5%)	0.001
Pain, prevalence	254 (73.0%)	240 (69.8%)	0.36
Pain assessment	184 (54.1%)	116 (34.3%)	<0.001
Prescribed rescue medication for pain	344 (98.3%)	317 (90.3%)	<0.001
Pain alleviated			0.74
Totally	213 (80.4%)	199 (81.6%)	
Partly	52 (19.6%)	45 (18.4%)	
Not at all	0	0	
Rattle, prevalence	204 (58.3%)	183 (53.5%)	0.206
Prescribed rescue medication for rattle	336 (96.3%)	316 (89.8%)	0.001
Rattle alleviated			0.88
Totally	113 (52.8%)	98 (52.4%)	
Partly	90 (42.1%)	84 (44.9%)	
Not at all	11 (5.1%)	5 (2.7%)	
Nausea, prevalence	45 (13.0%)	47 (14.2%)	0.21
Prescribed rescue medication for nausea	324 (92.6%)	259 (74.0%)	<0.001
Nausea alleviated			0.117
Totally	41 (69.5%)	27 (52.9%)	
Partly	15 (25.4%)	23 (45.1%)	
Not at all	3 (5.1%)	1 (2.0%)	
Anxiety, prevalence	194 (57.2%)	201 (62.2%)	0.19
Prescribed rescue medication for anxiety	344 (98.3%)	311 (88.4%)	<0.001
Anxiety alleviated			0.49
Totally	142 (68.6%)	134 (65.4%)	
Partly	65 (31.4%)	71 (34.6%)	
Not at all	0	0	
Dyspnea, prevalence	179 (52.2%)	150 (44.9%)	0.058
Dyspnea alleviated			0.060
Totally	83 (43.7%)	48 (31.4%)	
Partly	104 (54.7%)	101 (66.0%)	
Not at all	3 (1.6%)	4 (2.6%)	
Delirium, prevalence	77 (22.7%)	85 (26.5%)	0.26
Delirium alleviated			0.59
Totally	30 (33.0%)	23 (26.1%)	
Partly	49 (53.8%)	51 (58.0%)	
Not at all	12 (13.2%)	14 (15.9%)	

There were, however, significant differences for other parameters and activities. Compared to NH patients, SPC patients were more likely to receive symptom assessments during their last week of life as regards mouth health (*p* < 0.001), pain (*p* < 0.001), and assessment of symptoms other than pain (*p* < 0.001). They were also more likely to have prescriptions of rescue medications for pain (opioids), nausea (mainly metoclopramide or haloperidol), anxiety (benzodiazepines), and death rattle (anti-cholinergic medication, e.g., glycopyrronium bromide) (*p* < 0.001).

### Characteristics and content of care: psychosocial and existential aspects

According to the health care staff´s own registrations, death was, to a larger extent, expected more in SPC than in NH (98.3% vs 84.5%, *p* < 0.001) and SPC was more often the preferred place of death according to the patients (77.2% vs 62.0%, respectively, *p* < 0.001). EOL discussions were, to larger extent, held in SPC (88.1 vs 66.2%, *p* < 0.001), and dialogues and EOL discussions with relatives were more often held in SPC, both about changes in care and transition to EOL palliative care (*p* < 0.001, Table 3). Relatives in SPC settings were more often present at the time of death (65.3% vs 41%, respectively). They were also offered bereavement follow-up more often. Health care staff at SPC units were more satisfied with the care they had been able to provide.

**Table 3.** Characteristics of palliative care registered in specialized palliative care and in nursing homes. (chi-square).

Palliative care characteristics	Patients receiving specialized palliative care (*n* = 355)	Nursing home and short-term facilities (*n* = 415)	*p*-value, diff between sp pall care and nursing home
Death was expected	349 (98.3%)	337 (84.5%)	<0.001
Could the patient express his/her wishes?			<0.001
Yes, all the time	44 (12.7%)	61 (18.2%)	
Lost ability express hours before death	146 (42.2%)	102 (30.4%)	
Lost ability express days before death	143 (41.3%)	119 (35.5%)	
Lost ability to express weeks before death	12 (3.5%)	27 (8.1%)	
Lost ability to express months before death	1 (0.3%)	26 (7.8%)	
(EOL) communication	280 (88.1%)	210 (66.2%)	<0.001
Patients’ preferred place of death?			<0.001
Yes	234 (77.7%)	176 (62.0%)	
Do not know	50 (16.6%)	93 (32.7%)	
No	17 (5.6%)	15 (5.3%)	
Anyone present at time of death			<0.001
None	68 (19.2%)	88 (21.2%)	
Relatives	169 (47.6%)	109 (26.3%)	
Relatives and HCP	63 (17.7%)	61 (14.7%)	
HCP	52 (14.6%)	153 (36.9%)	
Do not know	3 (0.8%)	4 (1.0%)	
(EOL) communication with relatives/family			<0.001
Have no relatives			
Yes	3 (0.9%)	8 (2.3%)	
No	297 (88.4%)	242 (69.3%)	
Do not know	27 (8.0%)	73 (20.9%)	
Family invited to bereavement dialogue	9 (2.7%)	26 (7.4%)	<0.001
Yes	329 (93.5%)	271 (66.6%)	
No	10 (2.8%)	74 (18.2%)	
Do not know	13 (3.7%)	62 (15.2%)	
Parenteral or enteral infusion of fluids last 24 hours			0.65
No	314 (93.5%)	336 (96.3%)	
Yes	21 (6.3%)	9 (2.6%)	
Do not know	1 (0.3%)	4 (1.1%)	
Consultation with pain unit	2 (0.6%)	4 (1.0%)	0.53
Consultation with palliative care team	17 (4.8%)	37 (8.9%)	0.025
Consultation with other hospital unit	12 (3.4%)	22 (5.3%)	0.20
Contact with physiotherapists, occupational therapists and social workers	4 (1.1%)	10 (2.4%)	0.18
Spiritual counselor	5 (1.4%)	2 (0.5%)	0.18

### Predictors of place of care

In the bivariate logistic regression analyses the following variables became predictors with *p* < 0.20: age (*p* < 0.001), social situation (*p* < 0.001), BMI (*p* = 0.015), exercise capacity (*p* = 0.013), smoking (*p* = 0.001 and *p* < 0.001), heart failure (*p* < 0.001), ischemic heart disease (*p* < 0.001), hypertension (*p* < 0.001), diabetes (*p* = 0.001), osteoporosis (*p* = 0.026), and depression/anxiety (*p* = 0.036) (Table 4). In the multivariate stepwise logistic regression analysis, younger age and co-habiting increased the probability of dying in SPC.

**Table 4.** Logistic regression with nursing home or specialized palliative care as place of death as dependent variable. Nursing home as reference variable, i.e. 0 and specialized palliative care as 1.

	Specialized palliative care as place of death compared to nursing home	
	Bivariate	Multivariate
Independent variables	OR (95% CI) *p*	OR (95% CI) *p*
Constant		1171.14 *p* < 0.001
Time between last visit and death	1.00 (1.00, 1.00) 0.24	
Age, divided by 10 years	0.42 (0.34, 0.51) <0.001	0.36 (0.22, 0.61) <0.001
Gender (1= man, 2 = woman)	0.93 (0.70, 1.23) 0.61	
Social situation (0= living alone; 1= co-habiting)	2.67 (1.62, 4.40) <0.001	4.57 (2.17, 9.62) <0.001
FEV_1_% of predicted value	1.00 (0.99, 1.01) 0.99	
Number of exacerbations in the last 12 months	1.03 (0.95, 1.12) 0.47	
Number of hospitalizations in the last 12 months	0.94 (0.84, 1.06) 0.32	
BMI	1.02 (0.99, 1.06) 0.15	
Exercise capacity	1.09 (1.02, 1.17) 0.013	
Smoking:		
Non-smoker	1	
Have quit smoking	2.91 (1.56, 5.44) 0.001	
Still smoking	3.77 (1.93, 7.34) <0.001	
Quality of life (CCQ) (0-6)	1.00 (0.95, 1.06) 0.90	
Dyspnea (mMRC) (0-4)	1	
	0.98 (0.38, 2.51) 0.97	
	0.96 (0.37, 2.49) 0.94	
	0.58 (0.23, 1.44) 0.24	
	0.72 (0.30, 1.76) 0.48	
Comorbidity		
Heart failure	0.39 (0.26, 0.59) <0.001	
Ischemic heart disease	0.49, (0.33, 0.72) <0.001	
Stroke	0.75 (0.38, 1.45) 0.39	
Hypertension	0.54 (0.38, 0.76) <0.001	
Diabetes	0.47 (0.30, 0.74) 0.001	
Osteoporosis	0.59 (0.37, 0.94) 0.026	
Depression/anxiety	0.67 (0.46, 0.98) 0.036	
Lung cancer	1.08 (0.54, 2.12) 0.84	

## Discussion

Despite similar prevalence of symptoms, some patients were referred to SPC, whereas others were admitted to NHs. A higher mean age for persons residing in NHs, when compared to patients cared for at SPC, is a common finding, as there is a tendency to refer frail older patients with multiple illnesses, to geriatric services or to NHs.

However, if aiming at equal and equitable care, the main focus should be on actual care needs. In our register study, the symptom prevalence was similar in SPC and NHs. Moreover, NH patients had a higher prevalence of comorbidities, including cardio-vascular diseases. Regardless, they were admitted to NHs, which constitute a lower level of care in Sweden. Most of the staff at SPC units in Sweden are registered nurses or physicians, whereas a majority of the staff at NHs are assistant nurses. Our results support the results from Cohen et al., who analyzed place of death for patients diagnosed with lung cancer and patients diagnosed with COPD in fourteen countries (23). Compared to patients diagnosed with lung cancer, patients affected by COPD were less likely to die at home or in a specialist palliative care facility, but were more likely to die in hospital or to be referred to NHs (23).

The reasons for the possible inequality as regards referral to SPC cannot be revealed from our register study. Although we have measurements of symptom *prevalence*, we do lack data on symptom *severity and complexity*, which are important factors. One possible reason for the differences that we found might be that patients affected by more intense symptoms, or symptoms that were more difficult to alleviate, were admitted to SPC. As the proportion of relieved symptoms was similar in SPC and NH despite a great difference in the formal level of competence, this might be an indication of a difference in the initial symptom intensity and complexity. If so, the patients were referred to an adequate level of care. However, another explanation might be that older, frail people with multiple illnesses are routinely referred to NHs, without sufficient consideration of the actual symptom burden (24). In a qualitative study, barriers to admitting COPD patients to palliative care were, among others, found to be that exacerbations and death in COPD are unpredictable, that professional caregivers lack a coherent and proactive plan and have insufficient experience, and that they hold a negative view of palliative care for end-stage COPD (25). Moreover, there may be a vague or insufficient communication between patients and professional caregivers about care possibilities for end-stage COPD (25). This, together with our findings, indicate that there is a need to discuss palliative care with patients and relatives before routinely admitting patients to NH.

Although symptom prevalence and the proportion of alleviated symptoms were similar, there was a great difference in awareness and in use of a holistic palliative care approach as recommended in the WHO definition of palliative care (26). The staff´s awareness of the impending death was higher in the SPC units, which translated into pre-emptive measures, not only in symptom management, but also as regards psychosocial and existential domains. PRN rescue medications for pain, nausea, anxiety, and death rattles were generously prescribed for patients at SPC units. Moreover, EOL discussions with patients and relatives were performed to a higher extent. For this reason, it is likely that the relatives were more prepared of the impending death, were present when their loved ones died, and were offered bereavement follow-up meetings more frequently.

This is perhaps the greatest difference in the care delivered: whereas traditional care is focused on day-to-day care, the palliative care approach and the palliative care philosophy underline the importance of preparing for a good death. Not only in physical terms, but also as regards the psychological, social, and existential/spiritual domains, both for the patient and his or her family (27).

The results of our study indicate that health care staff should make informed decisions when referring patients with diagnosis other than cancer to EOL care. Despite having a similar prevalence of symptoms but a higher proportion of co-morbidities, older patients were still referred to NHs.

Judging from the frequency of successful symptom relief, NHs probably constitute an adequate level of care for a proportion of elderly COPD patients. However, the palliative care approach, including a higher awareness, would probably further increase their outcomes. If a conscious palliative care philosophy is also applied at NHs for patients who have prognostic uncertainty, symptom assessment of common symptoms, as well as prescription of rescue medication for frequent symptoms would be more likely, as a result. Moreover, it would be possible to initiate advance care planning and EOL discussions at an earlier stage, which would likely result in well-informed and more prepared patients. This would be an example of effective resource utilization.

Limitations to our study include its retrospective approach. The strengths are that the Swedish National Airway Register includes a representative number of patients diagnosed with COPD, with different severities and about two-thirds of deaths in Sweden are registered in the SRPC. However, merging two registers resulted in a substantial number of missing participants. Increasing the number of patients registered in both registers will improve the data quality in subsequent studies.

We conclude that despite similar symptom prevalence, older persons are more likely to be referred to NHs. If applying a palliative care philosophy in NHs, routine symptom assessment and prescription of rescue medication for frequent symptoms, would be more likely. Promoting advance care planning and EOL discussions at an earlier stage would result in more prepared patients and families.

## Disclosure statement

No potential conflict of interest was reported by the authors.
